# Phagedæna—Is It a New Disease?

**Published:** 1881-07

**Authors:** 


PHAGEDÆNA—IS IT A NEW DISEASE?—We call attention to the very interesting description of the disease which has been epidemic among the horses of Cincinnati, Chicago, and other Western cities. Dr. Bowler is inclined to think that it is a new disease. It does not seem necessary to suppose that there is any new specific infection acting as a cause.

